# Recording Postprandial Glucose Reactions with Potato Starch Structural Improvements

**DOI:** 10.1155/2023/1263896

**Published:** 2023-12-13

**Authors:** Sadaf Bashir, Zubair Farooq, Sobia Zafar, Tabussam Tufail, Huma Bader Ul Ain, Muzzamal Hussain, Entessar Al Jbawi, Purabi Saha, Roshan Kumar, Richard Owusu Nyarko

**Affiliations:** ^1^Department of Food Science and Human Nutrition, University of Veterinary and Animal Sciences, Lahore, Pakistan; ^2^Services Hospital, Jail Road, Lahore, Pakistan; ^3^University Institute of Diet & Nutritional Sciences, The University of Lahore, Lahore, Pakistan; ^4^Department of Food Sciences, Government College University Faisalabad, Pakistan; ^5^Agricultural Extension Directorate, MAAR, Damascus, Syria; ^6^Department of Pharmaceutical Chemistry, ISF College of Pharmacy, Moga, Punjab, India; ^7^Department of Pharmacy, Shree Dev Bhoomi Institute of Education Science and Technology, Dehradun, Uttarakhand, India; ^8^School of Medicine American International University of West Africa, Gambia

## Abstract

The current study is aimed at modifying the structural makeup of potato starch through the application of heat and moisture to better control the postprandial glycemic response. Heat-moisture treatment (HMT) was used to prepare potato starch using different moisture levels and temperatures. The samples were digested with pancreatin to determine the % of easily digestible, slowly digestible, and resistant starch. Subjects were given pudding made with HMT potato starch, and their postprandial glycemic response was tracked by measuring their blood glucose levels. In addition, incremental and total incremental areas under the curve (IAUC, TIAUC) were also assessed. The current findings of in vitro enzymatic digestibility of potato starch showed inconsistent results as measured at different time intervals. Adding moisture and heating the mixture to 30 and 70°C both increased the amount of rapidly digestible starch in all of the treatments from 20 to 40%. The maximum value of slowly digestible starch was 43.63% when the sample was heated to 30°C with a moisture content of 30%. The highest value (68.46%) for resistant starch was achieved at 20% moisture level and 30°C. After eating pudding, blood sugar spiked for the first 60 and 90 min before gradually dropping off over the next 240 min. As a whole, the highest IAUC and TIAUC values, as well as the glycemic index and load, were observed in potato starch heated to 70°C, which contained 40% moisture. Most parameters achieved their highest values when 40% moisture was added and the heat was applied at 70°C.

## 1. Introduction

Aside from wheat, rice, and corn, the potato (Solanum tuberosum) is the world's fourth most important food crop, with an annual global production of almost 380 million tonnes [1]. Potatoes have several nutritional properties, including high-quality protein and complex carbohydrates, most of which are starch, but which contain compressed particles that prevent gastrointestinal enzyme absorption [2]. Potato starch is distinguished from other commercially available starches by its large granule size, purity, amylose and amylopectin chain lengths, occurrence of phosphate ester collections on amylopectin, ability to interchange certain cations with consistent effects on viscosity behavior, and ability to form thick viscoelastic gels upon heating and successive cooling, all of which are cultivar and environment dependent [3, 4]. Amylose and amylopectin units connected by -1,4 and -1,6 glucosidic connections make up the bulk of starch's 100 m molecule. Amorphous amylose (25% by weight) is composed of linear chains of -1,4-linked glucose units. With a strength of -1,4 and -1,6 branch points comprising 4-5% of the total, amylopectin is a semicrystalline, very branched polysaccharide that makes up around 75% of the substance by weight [5]. Blood glucose level primarily depends upon individual characteristics like type and quantity of food intake, physical activity, and body's metabolic response [6]. Preanalytical factors that affect blood glucose level are smoking, caffeinated drinks, use of hypoglycemic drugs, heavy exercise, anxiety, and delay in sample processing. Careful attention to these modifiable factors by both clinician and laboratory staff is essential to ensure accurate glucose measurement. The organ that is responsible for the fasting value of blood glucose is the liver whereas it is the pancreas that is responsible for postprandial blood glucose (PPBG) value. Several hours after dinner, blood glucose level drops leading to a decrease in insulin level and a rise in glucagon level. Glucagon is responsible for maintaining adequate blood glucose levels in fasting conditions via the activation of metabolic pathways like gluconeogenesis and glycogenolysis in the liver. Higher FBG level is due to an increase in glucagon to insulin ratio as seen in diabetes, where the liver is involved in excess glycogen breakdown and gluconeogenesis. Despite having sufficient insulin, an individual may have a higher FBG value which is mainly due to insulin resistance which is the commonest cause of impaired fasting glucose tolerance and diabetes mellitus.

There are two types of starch: digestible starch and indigestible resistant starch (RS). This type of starch can be used as a substrate in the colon, where it will be fermented into short-chain fatty acids with serious consequences for human health. Starch's sensitivity to digestion is a valuable feature that is of particular concern for nutrition. Starch has been categorized into two types based on its digestibility: quickly digestible starch (RDS), which can be completely hydrolyzed after 20 minutes of incubation, and slowly digestible starch (SDS), which can be digested anywhere from 20 minutes to 120 minutes [7]. The levels of SDS and RS released have significant consequences on human health [8, 9]. Slow glucose release into the bloodstream affects postprandial glycemic and insulin responses when starch is not immediately digested by human digestive enzymes in the upper stomach. Low glycemic index foods, such as those associated with SDS and RS, play an important role in the prevention and treatment of many disorders. Potential health benefits of SDS are associated with its ability to maintain glucose metabolism and hence facilitate diabetic treatment. Postmeal responses and glycemic responses are affected when the starch composition of meals is altered by different cooking techniques [10, 11]. Physical, chemical, and enzymatic processes are widely used to reform starches to stimulate specific beneficial qualities. Starch that has been physically transformed through the application of moisture, heat, shear, or energy has won over a larger audience because it has no chemical reagent byproducts. Starch can be physically altered to change its physicochemical properties using a process called heat-moisture treatment (HMT), but its granular structure is preserved. The starch-to-moisture ratio, temperature, and heating duration are critical variables in hydrothermal processing. In high-temperature milling (HMT), starch is heated past its gelatinization point but is not given enough moisture to fully gelatinize. When starches are heated past their gelatinization temperature, their granular, lamellar, crystalline, and double-helical organization is instantly destroyed. The gelatinization temperature of individual starch granules varies when potato starch is cooked in additional water [12, 13]. As the temperature rises, the lowest-gelatinization-temperature grains begin to expand and become disordered, and the remaining entire grains are melted as a control. Gelatinized potato starch is easily absorbed by the body [14]. In terms of their morphology, structure, viscosity, and physicochemical qualities, starches are affected by HMT. Additionally, HMT improves the enzymatic digestibility of the treated starches, increasing the proportion of fast and slowly digestible starches while reducing the proportion of resistant starch [15, 16].

The purpose of this study was to maximize the glycemic response to modified potato starch. In this study, we identified different potato starch fractions according to their digestibility and glycemic impact in healthy individuals.

## 2. Methods

### 2.1. Dry Starch Content Analysis and Potato Starch Solution Production

To find out how much water was in the potato starch, we used procedure 44-19 [17]. Potato starch was dried in a hot air oven after being placed in a prepared, weighted metal dish. Weight reduction was determined as a function of the starch's moisture content using the following formula.

(1) $$\begin{array}{c} \text{Moisture content}\%=\frac{W1-W2}{\text{weight of sample}}\times 100, \end{array}$$

where *W*1 is the weight of starch sample and metal dish before heating and *W*2 is the weight of starch sample and metal dish after heating.

Moisture content in the sample was brought to different levels by adding distilled water by using the following formula:

(2) $$\begin{array}{c} C_{1}V_{1}=C_{2}V_{2}. \end{array}$$

Based on their moisture level, potato starch samples were created with a 20%, 30%, and 40% moisture content. After collecting these samples, we stored them in sealed glass containers for 24 hours at room temperature.

### 2.2. Heat-Moisture Treatments (HMTs)

The starch samples were gelatinized using HMT. To test the effects of each moisture level, the aforementioned potato starch solutions were made in airtight glass containers. Except for the control treatment, all of the produced samples were heated at either 30 or 700 degrees Celsius. For later use, all beakers were wrapped in aluminum foil and placed in the freezer.

### 2.3. Lyophilization

All samples were lyophilized in lyophilizer (-70°C) for 48 hours including control samples.

### 2.4. Powder Preparation

All the samples were ground and then sieved with a 100 mesh screen after drying.

### 2.5. Measurement of Starch Digestibility

For 10 minutes, 1 gram of pancreatin was mixed with 12 milliliters of distilled water using a magnetic stirrer. After adding pancreatin to the solution, we centrifuged it (1500g for 10 minutes). Ten milliliters (10 mL) of the murky supernatant was poured into a conical flask. As a means of gauging how easily hydrothermally processed starch can be digested, a microtube (2 mL) containing starch was mixed with sodium acetate buffer (0.1 M, pH 5.2, 0.75 mL) and a glass bead (30 mg, wet basis). After adding the enzyme solution (0.75 mL), the sample was shaken for 240 minutes at 240 revolutions per minute (rpm). At 10, 20, 60, 120, and 240 minutes, the tubes were retrieved, heated to halt the reaction, and centrifuged (5000g, 5 minutes). Each sample went through this process. Using a GO-POD kit, we determined the glucose concentration in the supernatant.  Table 1 shows the preparation of samples to measure glucose content in starch.

Each sample required three test tubes: a “blank,” “standard,” and “sample.” Each sample's optical density (O.D.) was then measured using a spectrophotometer set at 510 nm. The following equation was used to determine glucose concentrations:

(3) $$\begin{array}{c} \text{Glucose }\left(\mu \text{g}/0.1\text{ mL}\right)=\frac{\text{sample}}{\text{glucose standard}}\times 100. \end{array}$$

Starch contents were measured by using the following formula:

(4) $$\begin{array}{c} \text{Starch content}=\text{O}.\text{D}\times 0.9. \end{array}$$

RDS, SDS, and RS contents were measured as the amount of glucose released after 10, between 10 and 240, and after 240 minutes of digestion, respectively.

### 2.6. In Vivo Glycemic Response of Various HMT-Treated Potato Starches

Anthropometric measurements to check the accuracy of the volunteers are specified in Table 2. All the volunteers were healthy individuals with varying ranges of their BMI (21.34 ± 1.46 kg/m^2^). Average scores for age, weight, and height of the volunteers were 24.5 ± 1.06, 58.5 ± 6.27, and 5.51 ± 0.17, respectively. Table 2 details the anthropometric measurements that were used to validate the accuracy of the volunteers. All of the participants had a body mass index (BMI) of 21.34 ± 1.46 kg/m^2^. Volunteers averaged 24.5 ± 1.06, 58.5 ± 6.27, and 5.51 ± 0.17 years old, respectively.

### 2.7. Preparation of Pudding

Potato starch pudding was prepared based on different HMT starches using the following recipe.

The ingredients of pudding for one subject were the following:

Potato starch = 62 g

Sugar = 24 g

Skimmed milk = 500 mL

Vanilla extract = 2 drops

Ingredients were properly weighed, homogeneously mixed, cooked for 5 minutes, and then cooled to room temperature.

### 2.8. Efficacy Study

Posters at regular locations, fliers, emails, and word of mouth all contributed to the recruitment of eight healthy women. Subject eligibility was based on age (20-30), health (optimal body mass index, ideal BMI, glucose fasting level, free of communicable diseases, and other health anomalies including hypertension, cardiovascular diseases, and diabetes), and other factors. There is no discrimination of ethnicity and race of participants in this study. All participants were informed of the study's procedures and requested to sign a consent form before their participation was officially recorded. Subjects' postfasting blood glucose levels were measured through finger prick with a glucose sensor (Accutrend; Roche Diagnostics Corporation; Indianapolis, IN, USA) [18, 19]. All of the patients were given 50 g of pudding prepared with both HMTs on alternating days. After eating, blood glucose levels were checked every 30 minutes for up to four hours. During the testing session, the participants were asked to refrain from eating. Nonetheless, they were able to get a sufficient supply of water. Prior to and following consumption of the experimental meal, blood samples were taken from each participant to measure their blood sugar levels.

Blood glucose data from all individuals for glucose and test food were used to determine glycemic index, mean postprandial glucose level, area under incremental curve, and total area under incremental curve at a number of time points. In the end, the collected information was analyzed.

### 2.9. Statistical Analysis

All measurements were taken in triplicate, and analysis of variance was done at a significance level of *p* > 0.05. The study design was a randomized complete block design (RCBD). Duncan's multiple range test evaluated the means to determine if there was a statistically significant difference (DMRt). Statistical calculations were performed with the help of Costat-2003 and Co-Hort (version 6.303).

## 3. Results

### 3.1. In Vitro Enzymatic Digestibility of Potato Starch

Table 3 displays the digestibility of several potato starch treatments. In this experiment, two types of controls were set up to allow for a more precise evaluation of the in vitro digestibility of potato starch. The absence of any heat and moisture treatment being applied to the potato starch constituted the “general control treatment.” After 10 minutes, the digestibility rate in the general control was 14.67 ± 0.052%, decreasing to 3.59 ± 0.136, 1.64 ± 0.121, 1.78 ± 0.148, and 3.41 ± 0.478% after 20, 60, 120, and 240 minutes. There were designated controls used for all the other procedures that involved applying heat and moisture to potato starch.

All treatments, excluding the control group, enhanced the digestibility rate (percentage) after 10 minutes. The digestibility rate of potato starch increased somewhat at both 30 and 700°C when 20% moisture was added, from 21.363 ± 0.105 to 22.37 ± 0.105%. The digestibility rate of potato starch reduced (15.490.136%) at 30°C, but increased (32.71 ± 0.665%) at 700°C when more moisture was added at a rate of 30%. For a moisture content of 40%, the first digestibility rate (%) was reduced (26.110.973%) after being subjected to heat at 30°C, and then, it was enhanced (69.32 ± 0.588%) after being subjected to heat at 70°C.

When the digestibility rates (percent) of potatoes were measured after 20 minutes and compared among different HMT and their respective control treatments, differences were observed. The digestibility rate increased (3.52%, 0.136%) at 30°C and decreased (1.32%, 0.136%) at 70°C when the moisture content was 20%. While the digestibility rate rose (10.19 ± 0.166%) for potato starch heated to 30°C, it dropped (5.2 ± 0.504%) when heated to 70°C due to the addition of 30% moisture. The first digestibility rate (%) dropped to 9.24 ± 0.175 and 2.15 ± 0.558% at 30°C and 70°C, respectively, for a 40% moisture content.

The digestibility rate (percent) of potato starch after 60 minutes was higher for all treatments than the general control treatment. Depending on the treatment, the digestibility rate of potato starch varies. This was seen when comparing different treatments to their respective control treatments. The digestibility rate was 2.670.065 at 30 degrees Celsius and 2.53 ± 0.065 at 70 degrees Celsius when the relative humidity was 20%. The digestibility rates at 30 and 70°C improved to 9.56 ± 0.091 and 6.13 ± 0.548%, respectively, for a moisture content of 30%. The digestibility rate dropped from 5.78 ± 0.163 to 3.61 ± 0.085% when heated to 30 and 70°C, respectively, at a moisture level of 40%.

All treatments except the general control (1.78 ± 0.148%) had a higher digestibility rate after 120 minutes. At 30% and 70°C, the digestibility rate dropped to 1.85 ± 0.166% and 2.22 ± 0.166%, respectively, for a moisture level of 20%. When compared to the 20% moisture level, the 30% moisture level resulted in a higher digestibility rate of 13.80 ± 0.028 and 5.79 ± 1.035% at 30 and 70°C, respectively. When heated to 30 and 70 degrees Celsius, the digestibility rate for 40% humidity dropped to 6.08 ± 0.077 and 3.11 ± 0.473%, respectively, similar to what was seen for 20% humidity.

All treatments save for the 20% moisture level heated at 30°C (2.13 ± 0.105%) increased the digestibility rate (%) after 240 minutes, compared to the general control treatment (3.41 ± 0.478%). The digestibility rate (percent) dropped for both 20 and 40% moisture levels compared to their respective control treatments, whereas it rose when heated to 30°C.

### 3.2. Digestibility Components of Various Potato Starch Treatments

**Figure 1:**
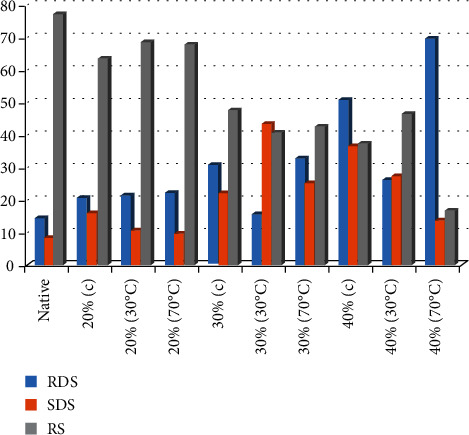
In vitro study of various components (RDS, SDS, and RS) of potato starch treated at different H-M treatments.

Rapidly digestible starch (RDS) is shown (Figure 1) at 10 minutes, slowly digestible starch (SDS) is shown at 20 minutes, and resistant starch (RS) is shown at 240 minutes, all of which are in vitro results of hydrothermally treated potato starches (SDS). All the treatments from 20 to 40% moisture addition heated at 30 and 70°C exhibited an increasing trend in fast-digested starch when compared to the general control treatment (14.67%). The RDS value was 15.49% lower for the treatment that was heated to 30 degrees Celsius at 30% relative humidity.

All the treatments had a wide range of SDS, although they were all relatively high compared to the control group's 8.43%. Maximum value was found at 30% moisture content heated at 30°C, and minimum value was found at 40% moisture level heated at 70°C (43.63% vs. 13.89%).

The highest value of resistant starch (RS) was found in native starch (control) at 77.23 percent. The potato starch with the highest value (68.46%) was treated with a heat-moisture combination of 20% moisture level heated at 30°C, while the potato starch with the lowest value (16.78%) was treated with 40% moisture level heated at 70°C.

### 3.3. In Vivo Glycemic Response of Various H-M-Treated Potato Starches

The glycemic response of 8 healthy individuals was checked by giving a pudding of potato starch. Potato starch was prepared by giving different moisture (20%, 30%, and 40%) and temperature (30°C, 70°C) treatments. After four hours of consumption of H-M-treated potato starch puddings, the results of starch treatment and time interval about the subjects were highly significant (*p* > 0.05).

Blood glucose concentration after consumption of pudding containing various H-M-treated starches initially increased and became highest at 60 and 90 min time interval, and after that, gradually, there was a decline till 240 min time interval. For the starch treatment containing 40% moisture level and heated at 30°C, the glycemic response was maximum at 60 min time interval which dropped gradually afterwards till 240 minutes. Similarly, for the starch treatment containing 40% moisture level and heated at 70°C, the glycemic response was maximum at 60 min time interval which stayed at the same level after 90 minutes and subsequently decreased till 240 minutes (results not shown).

### 3.4. Incremental Area under the Curve (IAUC) and Total Incremental Area under the Curve (TIAUC)

**Figure 2:**
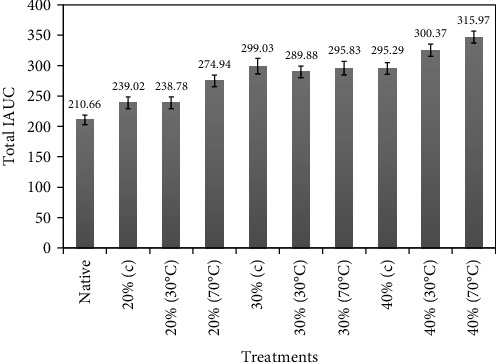
Mean ± standard errors of the total incremental area under the curve (TIAUC) for pudding prepared by various types of starches.

After giving the pudding (made of various treatments of heat-moisture-treated potato starches) to the healthy subjects, incremental area under curve (IAUC) and total incremental area under curve (TIAUC) were measured in terms of time intervals. The results of IAUC and TIAUC showed highly significant (*p* ≤ 0.05) results. For IAUC, all the results except for glucose (maximum value till 120 minutes), there appeared an increasing trend till 90 min time interval and then a steady decrease till 240 minutes. Overall, potato starch containing 40% moisture heated at 70°C had the maximum IAUC value (80.18 ± 14.78) followed by 76.06 ± 13.70 in the treatment containing 40% moisture heated at 30°C (results not shown). Similarly, TIAUC also displayed the same results where 40% moisture heated at 70°C had the maximum TIAUC value (315.97) followed by 300.37 for the treatment containing 40% moisture heated at 30°C. The native starch had minimum TIAUC value, i.e., 210.66 (Figure 2).

### 3.5. Glycemic Index

**Figure 3:**
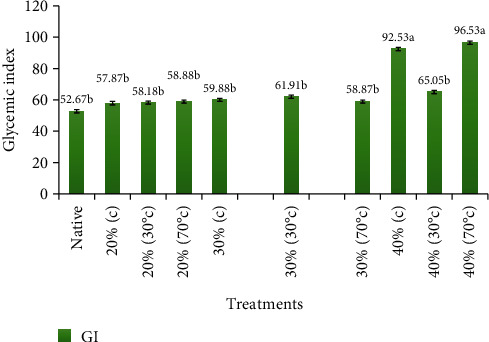
Mean ± standard errors of glycemic index (GI) for various types of pudding.

The highest score of glycemic index for pudding was 96.53 mmol/dL for the sample which had 40% moisture heated at 70°C followed by the treatment (65.05 mmol/dL) with 40% moisture heated at 30°C, and the native/control starch had the lowest glycemic index value, i.e., 52.67 mmol/dL (Figure 3). Analyses of variance showed nonsignificant results for the subjects while highly significant (*p* > 0.05) for the treatments/puddings made by various HMT.

### 3.6. Glycemic Load

**Figure 4:**
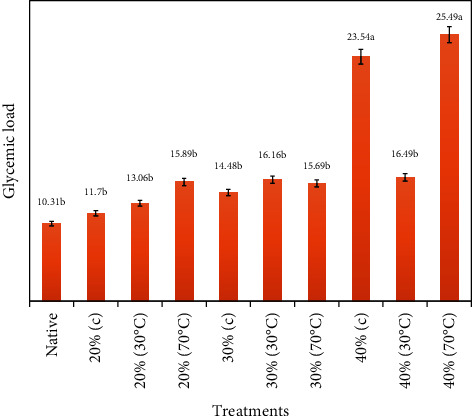
Mean ± standard errors of glycemic load (GL) for various types of puddings.

The highest score of glycemic index for pudding was 25.49 mmol/dL which was for the sample having 40% moisture heated at 70°C followed by the treatment (16.49 mmol/dL) with 40% moisture heated at 30°C, and the native/control starch had the lowest glycemic load value, i.e., 10.31 mmol/dL (Figure 4).

The analyses of variance (not shown) of glycemic load after four hours' consumption of pudding containing various H-M-treated starches regarding blocks (subjects) indicate statistically nonsignificant results (*p* > 0.05), but it was statistically significant concerning pudding types (*p* ≤ 0.05).

## 4. Discussion

Various researchers have reported different moisture percentages in potato starch [20, 21]. Moisture level in potato starch is the most important influencing factor in the formation of SDS which is obtained through HMT. HMT causes significant changes in the granular, crystalline, and aggregation structures of the potato starch [21, 22]. During HMT, there appears a new layer on the surface of starch granules which restricts water penetration into the granules, thereby retarding granule swelling [15, 23]. The induced changes depend upon the intensity of the treatment [24].

Enzymes play an important role in starch digestion and absorption. In the current study, the digestibility rate for various treatments of potato starch showed a wide variation. Uncooked starches like in the present study show negligible in vitro hydrolysis, whereas cooking for 10 minutes at 100°C increases both the rate and extent of hydrolysis of all the starches. Uncooked starches elicit no detectable glucose and insulin responses as compared to cooked starches. Chemical modification does not change the rate and extent of hydrolysis of the starches but rather the rate of absorption [25]. Due to enzymatic digestibility, some of the RS fraction changes into the SDS fraction.

Findings regarding SDS in this study are in close agreement with another study by Xie et al. [26] where the maximum yield of slowly digestible starch (SDS) in waxy potato starch reached 38.63% by retrogradation treatment under temperature cycles of 4/25°C for 3 days with an interval of 24 h. Similarly, another study of Lee et al. [11], explored that potato starch with 30% moisture content, heated at 30°C, produced the highest SDS content (37.5%). Hydrothermal changes in the potato starch increase enzyme susceptibility due to which the resultant modified product displays lower RS and higher SDS content.

Starch susceptibility during enzyme digestion is influenced not only by the hydrothermal levels but also by the storage condition. HMT intensity significantly influences SDS level as 41.8% SDS content has been reported to be obtained after 5 h 20 min at 120°C with a 25.7% moisture level. Induced structural changes in potato starch significantly affected digestibility and blood glucose levels [24]. Moreover, SDS decreases and RS content increases during potato starch modification through irradiation wherein RDS increases with increasing irradiation dose [27]. RS products might be useful substitutions for standard RDS to decrease postprandial glucose, and likely insulin, to better control blood glucose when incorporated into a food product [28].

Accordingly, in another study, the structural properties and digestibility of slowly digestible hydrothermally treated potato starch (SDS) with 30% moisture content, heated at 30°C, produced the highest SDS content (37.5%). Structural changes during hydrothermal treatment of potato starch significantly affect digestibility and blood glucose levels [11].

The results of the glycemic response of HMT potato starches are quite close to another study by Lee et al. [11], who reported that blood samples of mice were taken at 0, 30, 60, 90, 120, 150, 180, 210, and 240 minutes. Glucose responses for native starch having 20% moisture heated at 30°C, 30% moisture heated at 30°C, 40% moisture heated at 30°C, and amorphous starch samples were estimated to be 20, 62, 77, 88, and 91, respectively. Glucose, amorphous, and 40% moisture heated at 30°C samples caused dramatic increases at 30 min and then sharp decreases in the postprandial blood glucose levels. The starch having 20% moisture heated at 30°C and 30% moisture heated at 30°C samples displayed a much lower blood glucose level than the starch having 40% moisture heated at 30°C samples and amorphous starch sample due to a higher content of RS and SDS fractions. The decrease in blood glucose after 60 min was slower than that for the 40% moisture heated at 30°C sample. The starch having 30% moisture heated at 30°C samples and higher blood glucose levels at 60, 120, and 180 min was noticed as compared to the amorphous starch sample. This meant that the 30% moisture heated at 30°C sample contained a substantial amount of the slowly digestible fraction. The glucose response of the hydrothermally treated samples increased as the moisture level increased.

Starches with high-fat contents can change our appetite cycle because they can stay in the stomach for a longer period. Hence, meals would take time to empty stomachs which causes lower elevation in blood glucose levels because slow digestion demands less release of glucose by the liver into the blood [29].

Parada and Aguilera [7] profiled postprandial glycemic effect of potato starch which was heated at three different temperatures 57°C, 60°C, and 65°C for 10 minutes showing results for IAUC 38.0 ± 13.66, 130 ± 13.66, and 130 ± 13.66, respectively, which are also close to the findings of the current study. It means high temperature causes more increase in glycemic index. The incremental area under the curve falls with high RS (59%) which supports its insulin sensitivity consumption and postprandial plasma glucose [30]. Likewise, in another study by Haub et al. [28], the efficacy of four resistant starches was determined on postprandial glycemia and ratings of fullness. Blood glucose was measured in the fasted state following the randomly assigned treatments at 30, 45, 60, 90, and 120 minutes of postconsumption. A visual analog scale was used to determine fullness at each time point. There were no differences (*p* < 0.05) in the treatments and glucose incremental areas under the curve (IAUC) for certain treatments.

In terms of glycemic index and glycemic load, the starch sample with 40% moisture heated at 70°C had the highest score followed by the treatment with 40% moisture heated at 30°C. Native/control starch had the lowest values. Fluctuation in glycemic index for different individuals is due to the intra- and interindividual variability of glycemic index [31], but that does not necessarily mean that there are real differences between the subjects [32]. Many factors could lead to variations in blood glucose response even within the same individual.

The prevalence of high-grade liver steatosis increases significantly across quartiles of dietary GI [33]. Starches having high glycemic index are consumed and absorbed rapidly and can have a lethal influence on blood glucose balance. Blood glucose concentration (mmol/L) is doubled at least for 2 hours after consuming a high glycemic index meal than consuming a low caloric food item with less added sugar. Such starches have slow and sustained absorption, and thus, postprandial blood glucose levels go up and down in a slow manner without making sharp peaks and falls that are allied with a high glycemic index. The relation of GI to a range of discomforts and complications and raised blood glucose levels leads towards fat deposition, overweight issues, myocardial infractions, and insensitivity of nephrons. Once sugars have been absorbed into the blood stream, they are transported to the liver and built into glycogen as an energy store for the body. Excessive intake of foods will result in conversion to fat which can store more energy but cannot be released as quickly, so it may build up and become a health risk. Likewise, in vitro hydrolysis of starch and glycemic index for cooked potatoes has a significantly positive correlation [34].

## 5. Conclusion

The nutritional quality of potato starch has improved significantly after esterification by sodium trimetaphosphate. Furthermore, the material presents not only high digestion resistance but also similar gelatinization properties to its raw starch counterpart. Thus, potato starch phosphodiester has good application prospects in the functional foods and pharmaceutical industry. The application of HMT to the potato starch rendered quite variable effects regarding various parameters of evaluation, especially in vitro enzymatic digestibility, RDS, SDS, RS, and blood glucose concentration. The glycemic response was maximum after 60 minutes which dropped gradually afterwards till 240 minutes. Results for IAUC and TIAUC, glycemic index, and glycemic load showed maximum values for the potato starch treatment containing 40% moisture level heated at 70°C.

## Figures and Tables

**Table 1:** Preparation of samples to measure glucose content in starch.

Formulation	Reagent blank	Standard	Sample
GO-POD reagent	3.0 mL	3.0 mL	3.0 mL
D-Glucose standard	—	0.1 mL	—
Sample	—	—	0.1 mL
Buffer/water	0.1 mL	—	—
Total volume	3.1 mL	3.1 mL	3.1 mL

**Table 2:** Mean values of anthropometric measurements for volunteers.

Human subjects	Gender	Age (year)	Weight (kg)	Height (inches)	BMI (kg/m^2^)
1	Female	25	58	5.4	21.95
2	Female	25	54	5.4	20.43
3	Female	24	46	5.3	17.96
4	Female	25	65	5.8	21.79
5	Female	25	58	5.5	21.28
6	Female	26	60	5.7	20.71
7	Female	23	62	5.6	22.06
8	Female	23	65	5.4	24.60
Means ± SD		24.5 ± 1.06	58.5 ± 6.27	5.51 ± 0.17	21.34 ± 1.86

^∗^BMI = body mass index.

**Table 3:** In vitro digestibility rate (%) of various potato starch treatments at various time intervals.

Sample	10 min	20 min	60 min	120 min	240 min
Control	14.67 ± 0.052	3.59 ± 0.136	1.64 ± 0.121	1.78 ± 0.148	3.41 ± 0.478
20% c	20.82 ± 0.952	2.64 ± 0.360	4.25 ± 0.367	4.50 ± 0.595	4.57 ± 0.587
20% (30°C)	21.363 ± 0.105	3.52 ± 0.136	2.67 ± 0.065	1.85 ± 0.166	2.13 ± 0.105
20% (70°C)	22.37 ± 0.105	1.32 ± 0.136	2.53 ± 0.065	2.22 ± 0.166	3.77 ± 0.105
30% c	30.25 ± 0.509	6.40 ± 0.551	5.13 ± 0.119	5.66 ± 0.558	5.06 ± 0.491
30% (30°C)	15.49 ± 0.136	10.19 ± 0.166	9.56 ± 0.091	13.80 ± 0.028	10.07 ± 0.060
30% (70°C)	32.71 ± 0.665	5.2 ± 0.504	6.13 ± 0.548	5.79 ± 1.035	7.95 ± 0.479
40% c	50.92 ± 1.611	9.25 ± 0.077	9.53 ± 0.020	9.95 ± 0.076	6.81 ± 0.055
40% (30°C)	26.11 ± 0.973	9.24 ± 0.175	5.78 ± 0.163	6.08 ± 0.077	6.16 ± 0.100
40% (70°C)	69.32 ± 0.588	2.15 ± 0.558	3.61 ± 0.085	3.11 ± 0.473	5.01 ± 0.576

## Data Availability

The data that support the findings of this study are available on request from the corresponding authors.
